# Seaweeds in the Oncology Arena: Anti-Cancer Potential of Fucoidan as a Drug—A Review

**DOI:** 10.3390/molecules27186032

**Published:** 2022-09-16

**Authors:** Jun-O Jin, Dhananjay Yadav, Kajal Madhwani, Nidhi Puranik, Vishal Chavda, Minseok Song

**Affiliations:** 1 Shanghai Public Health Clinical Center & Institutes of Biomedical Sciences, Shanghai Medical College, Fudan University, Shanghai 201508, China; 2 Department of Microbiology, University of Ulsan College of Medicine, Seoul 05505, Korea; 3 Department of Life Sciences, Yeungnam University, Gyeongsan 38541, Korea; 4 Department of Microbiology, BETS Science College, Sabar Kantha 385535, India; 5 Department of Biochemistry & Genetics, Barkatullah University, Bhopal 462026, India; 6 Department of Pathology, Stanford School of Medicine, Stanford University Medical Center, Stanford, CA 94305, USA; 7 Department of Medicine, Multispeciality, Trauma and ICCU Center, Sardar Hospital, Ahmedabad 382350, India

**Keywords:** seaweeds, marine algae, marine drugs, fucoidan, sulfated polysaccharide, cancer, adjuvant, prebiotics

## Abstract

Marine natural products are a discerning arena to search for the future generation of medications to treat a spectrum of ailments. Meanwhile, cancer is becoming more ubiquitous over the world, and the likelihood of dying from it is rising. Surgery, radiation, and chemotherapy are the mainstays of cancer treatment worldwide, but their extensive side effects limit their curative effect. The quest for low-toxicity marine drugs to prevent and treat cancer is one of the current research priorities of researchers. Fucoidan, an algal sulfated polysaccharide, is a potent therapeutic lead candidate against cancer, signifying that far more research is needed. Fucoidan is a versatile, nontoxic marine-origin heteropolysaccharide that has received much attention due to its beneficial biological properties and safety. Fucoidan has been demonstrated to exhibit a variety of conventional bioactivities, such as antiviral, antioxidant, and immune-modulatory characteristics, and anticancer activity against a wide range of malignancies has also recently been discovered. Fucoidan inhibits tumorigenesis by prompting cell cycle arrest and apoptosis, blocking metastasis and angiogenesis, and modulating physiological signaling molecules. This review compiles the molecular and cellular aspects, immunomodulatory and anticancer actions of fucoidan as a natural marine anticancer agent. Specific fucoidan and membranaceous polysaccharides from *Ecklonia cava*, *Laminaria japonica*, *Fucus vesiculosus*, *Astragalus*, *Ascophyllum nodosum*, *Codium fragile* serving as potential anticancer marine drugs are discussed in this review.

## 1. Introduction

Modern pharmaceutical science is based on natural products (NPs) derived from cells and tissues of microorganisms, plants, and animals of both aquatic and terrestrial ecosystems. NPs are secondary or specialized metabolites employed as traditional medicines and are now regarded as pillars of conventional pharmacology. Secondary metabolites are critical prerequisites for competitiveness, stress tolerance, anti-predator adaption, anti-microbial immunity, anti-radiation, and marine anti-biofouling activities [1,2,3]. In order to overcome stress circumstances produced by fluctuating or variable environmental factors, for example, light intensity, temperature, moisture, and mechanical wounds, NPs are deemed important [4]. Moreover, NPs are critical modulators of complex interspecies interactions that have an impact on the survival and function of organisms [5]. As a result of the survival and environmental adaptation processes, very complex and diversified compounds that are extraordinarily potential therapeutics that synthetic small molecules cannot match are encouraged to be developed.

Although oceans cover more than three-quarters of the Earth’s surface and marine natural products (MNPs) have a pronounced cytotoxic activity, most bioactive secondary metabolites have been acquired from terrestrial organisms [6]. The adaptation to distinct environmental conditions has resulted in diversification and sophisticated development of marine creatures from their terrestrial counterparts. Marine flora is taxonomically diverse, chemically distinct, biologically efficient, and highly specialized. Most sessile marine invertebrates have been found to produce active natural chemical products for a variety of ecological functions, such as defense against predators, parasites, illnesses, and competition, making aquatic ecosystems an important potential source of bioactive chemical compounds [7]. Hundreds of novel MNPs and over 36,000 marine compounds are identified each year from micro and macro-organisms such as fungus, bacteria, microalgae, macroalgae, sponges, corals, and tunicates. Many of these compounds interact with receptors and enzymes, highlighting their pharmacologic activity and constantly attracting enormous attention in the biomedical area [8]. Several marine compounds have demonstrated the ability to intervene in complex inhibitory actions and regulate several physiological functions such as cell signaling, permeability, angiogenesis, and apoptosis [9]. In general, the marine environment has created several highly effective marine-derived compounds with potential anticancer effects confirmed in-vitro, in-vivo, and in clinical trials.

Cancer remains a major cause of morbidity and mortality in industrial nations despite decades of basic and clinical research on promising new medicines. Cancer development is a dynamic and long-term process involving many complicated components and follows a step-by-step progression that eventually leads to metastasis. Initiation, promotion, and progression are the three crucial steps in creating various forms of human cancer. After cardiac disorders, cancer is the second biggest cause of death in industrialized countries, accounting for one out of every four deaths. Cancer is a multifaceted disease caused primarily by acquired genetic changes that provide tumor cells an edge in terms of survival or proliferation [10]. It is a complex multi-step process involving multiple factors such as smoking, occupational exposure, environmental pollution, unreasonable diet, genetics, viral infection, etc. [11]. Self-sufficiency in growth signals, evading apoptosis, resistance to anti-growth signals, unbounded replicative potential, genome instability, unceasing angiogenesis, tissue invasion, and metastasis are the hallmarks of cancer. Research must therefore continue to improve existing therapies and create new cures. Chemotherapy, radiation therapy, surgery, and combinations of these therapies have all been used to treat cancer. Regrettably, this therapeutics has various negative effects and could be replaced by MNPs [12]. In MNPs, particularly marine drugs, have been at the forefront of the issue as a chemotherapeutic substitute due to several advantages such as abundancy, inertness, high structural flexibility, renewable nature, biodegradability, biocompatibility, bioavailability, non-toxicity, easy processing and extractability [13,14]. Epidemiological studies have recently revealed that marine chemicals significantly impact this proclivity. Some natural extracts have been discovered that target specific signaling pathways to prevent or slow down the carcinogenesis process at various stages and exhibit properties such as specificity, easy stimulation of cancer cell apoptosis and minimal cytotoxicity [15]. Hence, the search for minimal toxicity natural substances is the demand of current research priorities. The anticancer therapeutic aspect of fucoidan as a natural marine drug is discussed in this review.

### 1.1. Anti-Cancer Marine Drugs

Since cancer is one of the world’s deadliest diseases, the discovery of new anticancer drugs is extremely challenging. Recent improvements in marine bioprospecting have resulted in the identification of a large number of marine chemicals with anticancer potential [16,17]. However, whereas most marine compounds are obtained from shallow fauna, the latest anticancer compounds identified from deep sea species demonstrate how modern technology and deep sea sampling enable unequaled access to a previously untapped reservoir of biochemical diversity [18]. Only a few innovative synthetic compounds have become commercial treatments due to toxicity. Five anticancer drugs based on MNPs have previously been licensed for human use [19]. Most research into the anticancer capabilities of chemicals originating from marine invertebrates has focused on sponges, corals, and algae [20,21]. Complex ecosystems like coral reefs are characterized by severe competition for space and feeding pressure, which contain a high amount of bioactive metabolites. These metabolites have harmful or deterring effects and could be potent drugs against cancer. Various anticancer drugs are derivatives of these marine compounds that have been approved for clinical use, including cytarabine, vidarabine, nelarabine (prodrug of ara-G), fludarabine phosphate (prodrug of ara-A), trabectedin, eribulin mesylate, brentuximab vedotin, polatuzumab vedotin, enfortumab vedotin [22].

Marine plants are becoming more popular because of their ability to produce natural goods with outstanding health advantages. Marine algae is a rich source of vitamins, minerals, unsaturated fatty acid, polysaccharides and well-known bioactive compounds, including sulfated polysaccharides, phlorotannins and glycoproteins. Division Phaeophyta (known as brown seaweeds) includes the most studied species of marine plants, namely *Ecklonia*, *Laminaria*, *Undaria*, *Fucus*, *Astragalus*, *Codium*, *Asconodulum* and *Himanthalia* [23]. Seaweeds are abundant in active chemicals, like multi-ring sulphurouscyclics, macrolides and trace elements, as well as in polysaccharides, terpenoids, proteins, polyphenols, and sterols [24]. Brown algae, rich in sulfated polysaccharides and secondary plant metabolites, including fucoidans, phlorotannins, and fucoxanthin, can be used to find new medicinal agents [25,26,27,28]. These compounds have been proven to have anticancer capabilities in addition to antioxidant and immunomodulatory activities and favorable effects on chemotherapeutic side effects such as cardiovascular disease [29,30]. Antiproliferative and pro-apoptotic actions of fucoxanthin and phlorotannins have been documented [31,32]. However, the specific mechanisms have not been dismantled yet [33]. The latest research implies that the pure sulfated polysaccharides can be used as an anticancer fucoidan, which could aid in developing natural anticancer medications. Numerous studies have been undertaken to better understand the potential mechanism of fucoidan as a cancer-preventive drug in vitro and in experimental animal models and humans. There has been good documentation on the therapeutic potential of natural biodiversity along with its natural bioactive agents like as marine polysaccharides, especially fucoidan is present and this activity will enable us to develop new generations of cancer therapies [34].

### 1.2. Fucoidan

Fucoidan is a natural heteropolysaccharide that was isolated in 1913 by Kylin from sea brown algae, and he dubbed it “fucoidin” [35]. According to IUPAC standards, it is now known as “fucoidan”; however, it is also known as fucan, fucosan, or sulfated fucan [36]. The evolving term fucoidan covers a wide range of sulfated polysaccharides (FCSP) with a backbone of fucose (fucans), heterogeneous compositions (i.e., fucogalactans, xylofucoglucuromannan) and diverse origins [37]. Fucoidan has long been recognized as a therapeutic nutritional supplement in Asia due to its medicinal properties, including anticancer properties. Fucoidan has been a well-explored anticancer marine drug, with the first research findings appearing in the 1980s [38].

Fucoidan has been found to have several intriguing pharmacological properties, including antithrombotic, antitumoral, antiviral, and anti-inflammatory action (dos Santos Marlise A.) Animal research exploiting its numerous pharmacological characteristics has revealed anticancer and antimetastatic benefits [39]. Fucoidan delays cancer growth, exerts a cytotoxic effect, and manifests synergistic impact with anticancer chemotherapeutic drugs [40]. Over the last three years, published research on fucoidans has progressed, and a wide range of fucoidan extract products is now accessible. Fucoidans have recently been assessed, and their potential application as oncology treatments has been expanded [41]. The mechanism through which fucoidans can have a direct or indirect anticancer impact is becoming clearer. Anticancer activities of fucoidans include immune-modulatory and anti-inflammatory characteristics. Fucoidans’ capacity to bind to Toll-like receptors and intervene with the action of vascular endothelial growth factors (VEGF) and matrix metalloproteinases (MMPs) could explain their anti-neoplastic properties [42].

During the development of anticancer medications, it is critical to understand the mechanisms by which certain chemicals exert their effects. Angiogenesis suppression, cell cycle arrest, apoptosis and/or necrosis induction, and immunological activation are all tactics used by anticancer medications to limit tumor growth [43]. The mode of action of algae bioactive is largely determined by their composition and chemical characteristics. However, various brown algae species have different fucoidan structures and contents. 

#### Fucoidan Sources and Structure

Fucoidan is a water-soluble, sulfated, 1,2- or 1, 3- or 1,4-α-l-fucos polymer, found in brown marine algae as structural polysaccharides, accounting for 5–20% of dry algal weight [44,45]. Fucoidan is structurally diverse, containing fucose, uronic acid, galactose, xylose, arabinose, mannose, and glucose residues and various degrees of branching sulfate concentration, polydispersity, and irregular patterns of monomers [34,46]. The major sources of fucoidans are sea cucumber and brown algae. Brown seaweeds are a big selection of marine plants, including *Sargassum thunbergi*, *Ascophyllum nodosum*, *Fucus vesiculosus*, *Laminaria japonica*, *Fucus evanescens*, and *Laminaria cichorioides*, and so on, that are commonly found in diverse cold water sea areas (Saadaoui, Imen). Fucoidan is an anionic polysaccharide with a high concentration of l-fucose and sulfate ester groups. It is mostly derived from brown seaweed, but other algae species, such as *Fucus vesiculosus*, *Ascophyllum nodosum*, *Laminaria japonica*, and *Macrocystis pyrifera*, can be employed as sources, resulting in a variety of polymeric compositions [36,45].

Fucoidan molecular weight (MW) has been recorded in a wide range of sizes, ranging from 5–7 kDa to several hundred kDa. Low molecular weight fractions (LMWF), in particular, are thought to be more biocompatible [47]. The sugar content, glycosidic bonds, branching, and degree of sulfation and acetylation of fucoidan vary depending on the algal species [48]. *Laminaria japonica*, *Saccharina latissima*, *Sargassum polycystum*, and *Saccharina longicruris* predominantly contain galactose, while *Ascophyllum nodosum*, *Fucus serratus*, *Fucus vesiculosus* comprise glucose, mannose, xylose, and uronic acids as other monosaccharides [49,50,51]. The backbone structures of *Fucus vesiculosus*, *Fucus serratus*, and *Ascophyllum nodosum* are dominated by linear (1→3) and (1→4) glycosidic linkages, while *Chorda filum* has a complex backbone structure due to the addition of a few branched fucose residues to the main chains, and further *Analipus japonicus* and *Undaria pinnatifida* have highly complex and heavily branched backbone structures [51,52]. Exceptionally invertebrate fucans rarely have regular, repetitive patterns regarding sugar units, glycosidic linkages, branching points, sulfation, or acetylation [37]. However, sulfate content, degree of sulfation, and molecular weight are typically cited as factors influencing the bioactivities of fucoidan. Thus, due to structural variability and lack of regularity in fucoidan molecules, it is frequently difficult to formulate distinct conclusions about the chemical structures of fucoidans.

Fucoidan is a mucilaginous component from the brown algal surface. Different kinds of fucoidan can be obtained, and extraction procedures vary depending upon its source. In general, the extraction of fucoidan using dilute acid or alkalis takes a long time and a big volume of reagents. Chemical, physical, and/or enzymatic treatments and other purifying and fractionation techniques can be used to extract and purify fucoidans from seaweeds [27,53]. Moreover, microwave or ultrasound is utilized to create vibrations in cellular water molecules, breaking apart cells and enhancing the efficiency of the traditional water extraction process. Enzymatic extraction is a highly efficient and specific approach involving cell wall dissolution and cell content removal [26]. According to in vivo studies, anticancer activity exhibited by fucoidan is dependent on source, dosage, frequency, and mode of administration. As per current research, the metabolic rate of fucoidan is subjective to different methods of administration and concentration, which has typical effects on tumors [37].

## 2. Pharmacokinetics of Fucoidan

Several experimental activities have been carried out to address the in-vivo bioavailability so-called ADME studies of fucoidan, i.e., absorption, distribution, metabolism, and excretion. These fundamental pharmacokinetic factors aid in the development of dosing regimens. Experiments in lab rodents may reveal the absorption and bioavailability of fucoidan. In intestinal absorption studies on rats, *Fucus vesiculosus* fucoidan (737 kDa) was utilized. After treatment, a maximum concentration of fucoidan in serum was reached after 4 h, with residual absorbed fucoidan accumulating in the kidney. In addition, absorption of fucoidan from *C. okamuranus* in rats has also confirmed the accumulation of fucoidan in organs [54]. Furthermore, comparative bioavailability tests between LMWF and (moderate molecular weight fucoidan) MMWF have shown that LMWF has a better absorption rate and bioavailability; therefore, its biological potential is higher. Furthermore, in rats, following topical administration of fucoidan (MW 750 kDa) from *Fucus vesiculosus* demonstrated fine skin-penetrating characteristics. In comparison to an intravenous supply of the same dose, the topical use of the 100 mg/kg body weight resulted in a long half-life of the substance. Hence, fucoidan is taken up by endocytosis and has been detectable in human serum and urine during topical or oral fucoidan therapy [55]. Moreover, recent pharmacokinetic research of *Fucus vesiculosus* [56] confirmed that the MW of fucoidan plays a crucial role in its absorption and deposition, which could be linked to its prolonged half-life following topical application [57]. In addition, the pharmacokinetics of fucoidan may also be affected by the type of pharmaceutical formulation. According to Kimura et al., encapsulation of fucoidan in nanoparticles can boost at least partial cytotoxicity due to higher permeability. However, fucoidan’s pharmacokinetics with respect to toxicity may still be favorable; data on its biodistribution in humans is currently lacking. Few clinical trials are presently ongoing focusing on the bio-distribution and tolerance of fucoidan. The bio-distribution, safety, and dosimetry of a tagged fucoidan are investigated in healthy persons or volunteers (ClinicalTrials.gov, Identifier: NCT03422055). Patients with stage III-IV non-small cell lung cancer (NSCLC) are being evaluated in a placebo-controlled trial. Fucoidan is added to their chemotherapy treatment to see how it affects their malignancy and quality of life (ClinicalTrials.gov, Identifier: NCT03130829). The outcomes of these clinical trials are crucial in understanding ADME and fucoidan toxicity in humans. Although more research on absorption across the intestinal tract is needed, it’s fascinating to see scientists working on different aspects of fucoidan absorption. As a result of the massive amount of knowledge that is gradually becoming available, future developments of fucoidan as a drug will rely on well-informed decisions [58].

### 2.1. Fucoidan and Cancer

The anticancer mechanisms of fucoidan are multifaceted. Fucoidan has a broad range of impacts on cellular functions, including cell cycle regulation, RNA metabolism, protein metabolism, carbohydrate metabolism, bioenergetics, mitochondrial maintenance, and DNA repair pathways. Induction/inhibition of reactive oxygen species (ROS), mitochondrial instability, and caspase and poly (ADP-ribose) polymerase (PARP) cleavage are all aspects of it [59,60]. Increased radiation susceptibility [61], prolonged cell cycle arrest, improved immunological clearance, enhanced apoptosis, decreased DNA damage, and metastatic suppression seems to be direct and indirect anti-neoplastic actions of fucoidans. Fucoidans are independent checkpoint modulators that can be used with modern checkpoint inhibitors to treat cancer [62].

#### 2.1.1. Anticarcinogenic Mechanism of Fucoidan

According to previous studies, the anticancer mechanism of fucoidan largely covers four components. They are (i) cell cycle arrest, (ii) induction of apoptosis, (iii) anti-angiogenesis and (iv) anti-inflammatory activities. 

#### 2.1.2. Role of Fucoidan in Cell Cycle Arrest and Apoptosis

Fucoidan inhibits cancer cell proliferation by regulating the cell cycle and blocking uncontrolled mitosis. Alekseyenko et al., 2007, performed Lewis lung cancer transplantation in C57 mice and found that tumor mass and lung metastasization were significantly reduced in fucoidan-treated mice, showing that fucoidan successfully suppressed tumor cell metastasis and proliferation in vivo [63]. Fucoidan (22.5 mg/mL) therapy-induced cell cycle arrest in the G2/M phase and inhibited cell growth in HCC cell lines [64]. Fucoidan boosted the number of G1/S cells in HUT-102 and NSCLC-N6 cell lines, while fucoidan promoted apoptosis and cell cycle arrest in HTLV-1 infected T cells and MCF-7 cells [65,66].

Programmed cell death, such as apoptosis, necroptosis and autophagy, is normally the result of an imposed form of cell cycle arrest. Apoptosis is essential for the survival of multicellular organisms. In general, apoptosis is required for homeostasis and is frequently deregulated in human diseases such as cancer [67]. In contrast to necrosis, apoptotic cells experience chromatin condensation, DNA fragmentation, cleavage of specific proteins, cellular DNA damage, morphological alterations such as blebbing and the budding of apoptotic bodies [68], as well as distinct energy-dependent biochemical mechanisms [69]. Fucoidan promotes apoptosis in cancer cells by aggressively activating apoptotic signals and their underlying downstream pathways. Eun et al., 2010 cocultured human colon cancer cells HT-29 and HCT116 with fucoidan derived from *Fucus vesiculosus* [70]. Fucoidan caused caspase-3, -7, -8, -9 activation, chromatin condensation, and PARP cleavage. According to the findings, fucoidan can effectively cause apoptosis via caspase-8 and -9-dependent pathways. In human lymphoma HS-Sultan cell lines, fucoidan suppresses tumor growth and promotes apoptosis [71]. Similarly, in MCF-7 cells, fucoidan extract enhanced mitochondrial depolarization by upregulating proapoptotic proteins Bax and Bad and downregulating antiapoptotic proteins Bcl-2 and Bcl-xl expression [72]. Furthermore, fucoidan treatment causes PARP cleavage and caspase-3/7 activation in MCF-7 cells, which are hallmarks of apoptosis [73]. Interestingly, Miyamoto et al., 2009 reported that MCF-7 cells require caspase-7, whereas activation of caspase-3 is not necessary for fucoidan-induced apoptosis [74].

Fucoidan can also show an antitumor effect through simultaneous cell cycle inhibition and induction of cancer cell apoptosis. In the human hepatoma SMMC-7721 cells, fucoidan therapy caused noteworthy growth inhibition and ROS-mediated apoptosis involving several distinctive features such as chromatin condensation or marginalization, vacuolization, lower glutathione consumption (GSH), mitochondrial swelling, and depolarization of the mitochondrial membrane potential [75]. Fucoidan inhibits PI3K, suppressing ERK and activates MAPK, limiting cancer cell proliferation and decreasing Bcl-2 to Bax ratio, inducing caspase-dependent apoptosis in BEL-7402 and LM3 cell lines [76]. Fucoidan inhibited cell growth in hepatocellular, cholangiocarcinoma, and gallbladder carcinoma cell lines by inducing apoptosis and inhibiting cell cycle progression in a dose- and time-dependent manner [64].

#### 2.1.3. Fucoidan and Angiogenesis

Cancer cells rely on an adequate supply of oxygen and nutrients for cell proliferation and metastatic dissemination, highlighting angiogenesis and lymph angiogenesis are critical processes for tumorigenesis. Antiangiogenic therapy has proven to be an effective way to slow tumor growth; consequently, angiogenesis inhibitors are a popular target for cancer treatment [77]. Surplus sulfated fucoidan promotes antitumor and antiangiogenic actions in cancer cells. Fucoidan suppresses vascular endothelial growth factor (VEGF) expression, restricts angiogenesis, cuts off the neoplastic cells’ nutrients and oxygen, lowers its volume, and impedes invasion and metastasis. Using blocking VEGF165 from attaching to its cell surface receptor vascular endothelial growth factor receptor-2 (VEGFR-2), Koyanagi et al., 2003 observed that both natural and oversulfated fucoidans dramatically reduced the mitogenic and chemotactic activities of VEGF165 on HUVEC. They also claimed that increasing the number of sulfate groups in the fucoidan molecule increases its antiangiogenic and antitumor properties [78]. Tse-hung et al., 2015 supplied fucoidan to Lewis lung cancer cell implanted mice, resulting in reduced serum and lung tissue VEGF levels when compared to controls [79]. Accordingly, in mice implanted with Sarcoma-180 cells, both native and oversulfated fucoidans suppressed neovascularization [78]. Moreover, the experimental angiogenesis test showed that the expression of the angiogenesis factor VEGF-A was dramatically reduced by 100 mg/mL fucoidan [80]. Fucoidan-mediated in vivo inhibition of neovascularization is also reported [78]. These findings clearly state that fucoidan’s antitumor activity is attributable to its antiangiogenic effect. 

Fucoidans exhibit entirely diverse effects on angiogenesis depending on their molecular weight and sulfate content. Natural fucoidan with a high molecular weight (30 kDa) has antiangiogenic properties, inhibiting proliferation, migration, endothelic tube formation, and vascular network formation, whereas low (4–9 kDa) and intermediate (15–20 kDa) molecular weight fucoidan stimulate angiogenesis and enhance HUVEC migration, respectively. Contrastingly, over-sulfated fucoidan seems to have a more potent antiangiogenic property than natural or desulphated fucoidan [81].

#### 2.1.4. Fucoidan and Immunomodulation

Fucoidan remodels the immune system to improve the sustainability of cancer-specific T cells and NK cells and results in the elimination of cancer cells. Crude and modified fucoidans have been proven to have immunopotentiating effects in tumor-bearing mice, resulting in antitumor efficacy [27,53]. Several studies have confirmed crude fucoidan’s profound cell survival and natural killer activity in vitro and in vivo [82]. Farzaneh et al., 2015 found that fucoidan limits the development of promyelocytic leukemia cells in vitro and in vivo by boosting the cytotoxic activity of NK cells and delaying tumor formation [40]. In line with this, fucoidan reduced cell proliferation in Huh7 human liver cancer cells more effectively by inhibiting the chemotaxin CXCL12 and CXCR4 pathway, an effective target for cancer therapy [54]. Fucoidan has a strong anti-inflammatory activity; however, the underlying mechanisms exerted by fucoidan have not been fully elucidated. Fucoidan has been shown to impede lymphocyte adhesion and invasion, enzyme inhibition, and induce apoptosis at various phases of the inflammatory process. The downregulation of MAPK and NF-B signaling pathways, as well as selectins and the concomitant decrease in the generation of proinflammatory cytokines, is the most often debated putative mode of action for fucoidan [83,84].

#### 2.1.5. Fucoidan and Metastasis

**Figure 1:**
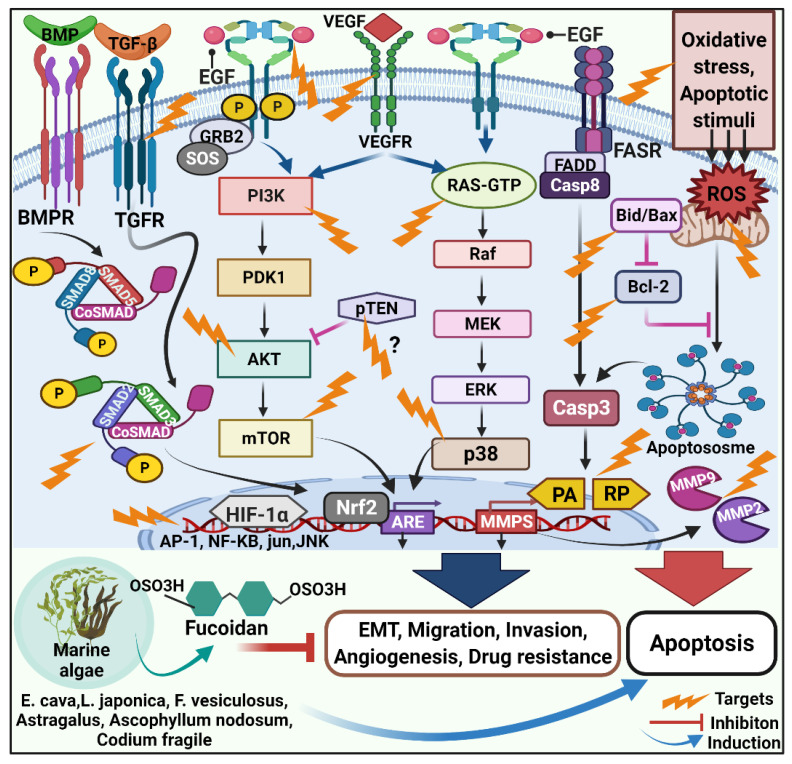
Schematic representation of following signaling pathways and molecules targeted by fucoidan to exert anticancer activities. Fucoidan from marine algae modulates TGF-β, PI3K/Akt/MAPK and extrinsic and intrinsic apoptosis pathways to enhance cytotoxic effects. The aforementioned pathways are deregulated in cancer, resulting in Epithelial-mesenchymal transition (EMT), migration, adhesion, drug resistance and metastasis. Fucoidan enhances antimetastatic effects, induces apoptosis, and serves as a potential marine anticancer drug.

Tumor cell proliferation and survival are frequently linked with deregulated intracellular signal transduction and persistent activation of cellular pathways [38]. Metastasis is a leading cause of cancer-related fatalities, accounting for up to 90% of all cases due to its systemic and pervasive nature and higher drug resistance. Metastasis is the multi-stage process through which cancer cells spread to different body areas through circulation, lymphatic system, or direct extension. The antimetastatic property of fucoidans is evidenced in both in-vitro and in-vivo studies. Fucoidan had moderate anticancer and antimetastatic effects following single and recurrent administration at a dose of 10 mg/kg [63]. Fucoidans from *L. saccharina*, *L. digitata*, *Fucus serratus*, *Fucus distichus*, and *Fucus vesiculosus* effectively inhibited MDA-MB-231 breast carcinoma cell adhesion, underlining anti-metastasis outcomes [84]. During metastasis, malignant cells break away from the primary tumor and invade nearby tissues by destroying the extracellular matrix (ECM) with MMPs. In a mice model of lung cancer, increased levels of fucoidan exhibited diminished MMP-2 activity and altered expression of adhesive and migratory proteins, such as integrin, VEGF-1 and -2, P-selectin and neuropilin-1 [85,86]. In the 4T1 xenograft model, fucoidan greatly lowers tumor volume and the number of metastatic lung nodules by minimizing cell migration and invasion while suppressing cell proliferation, colony formation, and expression of epithelial to mesenchymal transition (EMT) biomarkers in vitro [59,60]. An important function in the modulation of EMT in cancer cells is the molecular network of transforming growth factor-β (TGF-β) receptors (TGFRs). Fucoidan lowers the amount of TGF-RI and TGF-RII proteins, including Smad5/8 and Smad2/3 phosphorylation and Smad4 expression, affecting downstream molecular signaling. Fucoidan inhibits ECM degradation and reduces invasiveness in hepatoma HCC cells by downregulating the TGFR and SMAD signals [87]. Fucoidan also can efficiently reverse TGFRs, elicit EMT, stimulate the expression of epithelial markers, depress the expression of interstitial markers, and diminish the expression of transcriptional repressors, including Twist, Snail, and Slug. MAPK/ERK/PI3K/Akt/mTOR pathways regulate cell proliferation, differentiation, and death, and thereby the number of malignancies exploits all of them to be invasive or migratory. Antimetastatic effect of fucoidan is owing to its ability to block MAPK cascade, inactivate ERK1/2 pathway, inhibit phosphorylation of PI3K-Akt, and suppress mTOR and its downstream 4E-BP1 and p70S6K, and downregulate the expression of MMP-2 in time- and concentration-dependent manner. Remarkably, the highest concentration at which fucoidan has the most inhibitory effect is 200 mg/mL [88]. Fucoidan preferentially suppresses lung cancer by promoting Smurf2-dependent ubiquitination of TGF receptors [89], downregulation of MMPs, VEGF, and activation of tumor apoptotic signaling molecules. Besides this, fucoidan therapy also suppresses NF-kB and activator protein-1 (AP-1) transcription factors that are deregulated in cancer, inhibiting tumorigenesis, promotion, and metastasis [90,91]. AP-1 binds to the promoters of several cellular genes and starts their immediate gene expression as these genes are involved in the transcriptional regulation process. Fucoidan suppresses the AP-1 activation by inhibiting JunD expression in an HTLV-1-infected T-cell line, thereby inhibiting HTLV-1-infected T-cell proliferation [66]. Fucoidan inhibits Ik-Ba phosphorylation and raises total p65 in the cytoplasm while decreasing it in the nucleus, modulating inflammation, immunity, differentiation, cell growth, tumorigenesis and apoptosis [88]. Fucoidan inhibits the transcription of c-fos, c-jun, and AP-1 as well as decreases phosphorylation of ERK and JNK via inhibiting EGF-induced EGFR phosphorylation [92], enhancing antimetastatic activities (Figure 1). 

Furthermore, post-transcriptional mechanisms have been linked to TGF signaling via microRNAs (miRs) and have been involved in regulating EMT. Apparently, dose-dependent overexpression of microRNA-29b (miR-29b) in human HCC cells was demonstrated by blocking its downstream DNA methyltransferase 3B target (DNMT3B) and enhancing tumor metastasis suppressor gene 1 (MTSS1) expression [87]. Fucoidans are promising candidates being used as functional foods and pharmaceuticals for cancer prevention, chemotherapeutic agonists, and nanotechnology-based targeted therapy due to their extensive bioactivities in cancer, notably in metastasis.

#### 2.1.6. Fucoidan and Gut Flora

Due to their commercial abundance, brown seaweeds are frequently used as food additives. Fermentation of high molecular Fucoidan extracted from Ascophyllum nodosum and Laminaria japonica lowers inflammatory response while promoting Ruminococcaceae and Lactobacillus, respectively [93]. Meanwhile, dietary fucoidan progressively restores intestinal villi by upregulating the expression of tight junction proteins such as ZO-1, Occludin, Claudin-1, and Claudin-8 via p38 MAPK and ERK1/2 activation. Eventually, fucoidan supplementation improves intestinal barrier function by enhancing intestinal microbiota diversity and causing changes in microbial composition with a higher Bacteroidetes/Firmicutes phylum ratio. Fucoidan induces the enrichment of short-chain fatty acids producer Prevotella at the genus level [94]. A study revealed that fucoidan could be used as a gut flora modulator for breast cancer prevention. More detailed research is needed to better understand the association between cancer and intestinal flora and the anticancer mechanism of fucoidan [93]. As a result, the improvement of gut microbiota, as well as antiobesogenic, anti-diabetic, anti-microbial, and anticancer bioactivities offered by Fucoidan, underlines the need for more research to analyze the structure-dependent fermentation of fucoidan to assign a prebiotic effect. Table 1, Table 2, and Table 3 illustrates preclinical and clinical studies of fucoidan and its anticancer action mechanism.

## 3. Fucoidan from Different Sources of Marine Sea Weeds 

### 3.1. Ecklonia cava

*Ecklonia cava* (*E. cava*) is a brown alga widely distributed in China and Korea. *E. Cava* comprises abundant bio-active sulfated polysaccharide complexes. According to recent marine investigations, *E. cava* possess noteworthy antioxidant activity and is consequently competent in reducing the development of apoptotic bodies. Sulfated polysaccharide isolated from *E. cava* substantially promotes apoptosis via regulating pro-apoptotic caspases-7 and -8, Bax, and Bcl-xL [130]. According to Ahn et al., 2015 protamex extract (PCP) of *E. cava*, which is rich in fucose and sulfate components, strongly correlates with inhibitory growth sequels rather than antiproliferative effects involving enhanced apoptotic sub-G1 DNA contents, partial DNA fragmentation, hypo diploid sub-G0/1-peak as well as dosage dependent activated PARP and caspase 9 by dramatically modulating the Bcl-2/Bax signal pathway, indicating anticancer impact on colorectal carcinoma cells [68]. Additionally, U937 leukemic cells showed similar growth inhibitory results with sulfated polysaccharides of *E. cava* [131,132]. Furthermore, numerous researchers have discovered that fucoidan extracted from *E. cava* induces death in MCF-7 and HCT-15 cells via the activation of caspases and the regulation of MAPK, including ERK, p38 kinase, and the Akt pathway, as well as alterations in Bcl-2 and Bax [71,74,133]. Yet, it remains to be seen whether PCP is linked to MAPK signaling pathways such as ERK, p38 kinase, and the Akt pathway in CT 26 cells [68]. However, the efficiency of fucoidan in inducing apoptosis varied between different types of colon cancer cells.

Several preclinical trials have recently reported that *E. cava* fucoidan (ECF) has significant immunomodulatory effects. In view of this, ECF-mediated potent immunostimulative effects and cytotoxic effects on B16 and CT-26 carcinoma growth were reported in the mice model. However, the immunostimulatory effects of ECF on DC activation and anticancer immunity have been thoroughly investigated. Accordingly, ECF suppresses tumor growth by boosting DC activation and anti-inflammatory cytokine production. However, ECF promotes immune activation but has little effect on cancer cells since it is not an Ag-specific immune response. Because of this, cancer is treated differently from a bacterial infection in the body. ECF requires cancer antigens to stimulate an Ag-specific immunosuppressive response that targets tumors directly and promotes apoptosis to limit tumor growth. As a matter of fact, currently, cytotoxic T cell (CTL) activation is extensively exploited in cancer immunotherapy. Cancer antigen-specified CTL activation causes identification and apoptosis of antigen-presenting cells (APCs) through production of inflammatory cytokine and cytotoxic proteins such as perforins and granzyme B. CD8+ DCs activate antigen-specific CTLs in mice when antigens are presented on major histocompatibility complex (MHC) class I, which interacts with TCRs and CD8 on T cells [134,135]. Apart from antigenic presentation, CD8+ DCs produce a lot of co-stimulators and proinflammatory cytokines, which aid CD8 T cells in being differentiated into CTLs [136]. Antigen presentation, cytokine production, and co-stimulation are all signs of DC maturation. Moreover, in vitro activation of CTLs mediated by bone marrow-derived dendritic cells (BMDCs) is challenging to explain in mice, as BMDCs show distinct phenotypes and functions than in vivo DCs. In a mouse model, the combination of ECF and ovalbumin (OVA) administration resulted in ECF-mediated splenic CD8+ and CD8-DCs activation, enhanced OVA-specific T cell proliferation and IFN-γ production. These findings show that ECF, as an immunostimulatory molecule, significantly enhances antigen-specific immune responses in rodents [134]. Thus, ECF may be effective as an adjuvant to improve immune response against human malignancies. In a previous study, we compared the effects of four fucoidans isolated from *Ecklonia cava*, *Macrocystis pyrifera*, *Undaria pinnatifida*, and *Fucus vesiculosus* on human peripheral blood dendritic cells (PBDCs). In contrast to the other three fucoidans, we found that ECF significantly upregulated co-stimulatory molecules, MHC class I and II, and the production of proinflammatory cytokines in monocyte-derived DCs (MODCs) and PBDCs. Based on these findings, the study proposed that ECF may have the ability to increase human immunological activation [137].

### 3.2. Laminaria japonica

*Laminaria japonica* (*L. japonica*) is uncommon edible seaweed in Asia. *L. japonica* is a type of edible seaweed that is widely consumed in Asia. Laminarin is a polysaccharide composed of (13)-beta-d-glucan and (16)-beta-linkage that is isolated from *L. japonica* [138]. Laminaria has been shown to have antioxidant, antibacterial, anti-inflammatory and hypolipidemic properties. It has also been used as herbal medicine to treat cancer, gastrointestinal issues, diabetes, and arsenic poisoning. Preliminary trials suggested that *L. japonica* polysaccharides serve as a potent immunomodulator playing a pivotal role in combating cancer. In this context, polysaccharides from *L. japonica* can stimulate immune cells of both innate and adaptive immune systems, increasing the release of cytokines such as IL-10, IL-6, and IL-1 α, rendering them promising anticancer immunotherapy. It stimulates macrophages that directly release anticancer inflammatory molecules like NO and TNF- α while promoting bone marrow dendritic cells (BMDCs) maturation to activate naive T lymphocytes, thereby resulting in tumor cytotoxicity. Meng et al., 2014 confirmed declining phagocytosis, decreased lysosomal count, upregulated expression of BMDC’s major membrane components, including CD80, CD83, CD86, CD40, and MHC II, and increased BMDC-released production of IL-12 and TNF- α, all of which have been shown to significantly increase BMDC maturation by *L. japonica* polysaccharides [139]. Therefore, investigation of the immunological significance of laminarian indicated considerable BMDC maturation through increased expression levels of co-stimulatory molecules, IFN-γ and TNF-α cytokines production, and consequent cytotoxic T lymphocyte activation. As a result, it is reasonable to conclude that *L. japonica* polysaccharides have a high potential to boost BMDC maturation and can be employed as an immunological stimulant as well as a therapeutic adjuvant in immune-compromised individuals [139]. Furthermore, Lin et al., 2016 revealed that optimal fermentation products of *L. japonica* enhance anti-inflammatory effects by lowering ROS production, prostaglandin E2, NF-kB (p65) phosphorylation, and macrophagic NO production [140]. Fucoidan is a more powerful free radical scavenging antioxidant than laminarin due to the presence of the sulfate groups and the anionic charge [14]. As a result of these findings, laminarin has the potential to be employed as a novel immune stimulatory molecule for cancer immunotherapy.

### 3.3. Fucus vesiculosus

Fucoidan from *Fucus vesiculosus* (*F. vesiculosus*) induced apoptosis in HCT-15 cells by activating ERKs, p38, and inhibiting the PI3K/Akt signaling pathway [133]. Another study reported that *F. vesiculosus* fucoidans deplete cytosolic and nuclear NF-KB localization to suppress the invasion and migration of human lung cancer cells [88]. According to Ulf et al., 2015 treatment with *F. vesiculosus* resulted in a strong inhibition of viability in various pancreatic cancer cell lines. The fucoidan extract arrested the cell cycle at the S/G2 phase in cancerous proliferating cells due to the up-regulation of cell cycle inhibitors such as p21, p27, p57 and TP53INP1 while unaffecting non-malignant resting cells [141]. Hence, as a side effect of *F. vesiculosus* therapy, apoptosis was induced by PARP-cleavage in tumor cells rather than mitochondrial pathways. Intriguingly, in combination with autophagy inhibitors, enhanced cytotoxicity was reported. Overall, *F. vesiculosus* is a powerful cancer cell growth inhibitor; however, it neither causes mitochondrial inflammation nor causes caspase-dependent apoptosis, or necroptosis in normal cells, underlining the non-toxicity and antioxidant properties of fucoidan [141].

Researchers have been led to study the synergistic effects of fucoidan with the existing anticancer medicines. Doxorubicin (DOX) is renowned for its powerful dose-dependent anticancer efficacy, but a slew of significant side effects constrains it. Acute cardiotoxicity is a major side effect; hence, doxorubicin-loaded fucoidan nanoparticles are currently being investigated as a viable and safer option. Fucoidan, derived from *F. vesiculosus*, reduces doxorubicin-induced acute cardiotoxicity through modulating JAK2/STAT3-mediated apoptosis and autophagy [142]. By virtue of unique accumulation and preferential location in tumors, doxorubicin-loaded fucoidan nanoparticles demonstrated stronger anticancer activity in mice [143] or lower inhibitory concentration in cancer cells [144] compared to the free DOX model. Besides this, *F. vesiculosus* combined with paclitaxel had a potential antagonistic effect in breast cancer models, according to Burney et al., 2018 [145]. Moreover, recent in vivo human ovarian cancer murine model experiments illustrated no change in paclitaxel activity when given in combination with *F. vesiculosus*, contrary to prior in-vitro investigations. Additional studies are reasonable to delimitate mechanisms contributing to a discrepancy in the in vivo activity when given in combination with paclitaxel. As a preliminary stage, pharmacokinetic clinical research is presently underway to appraise the impact of fucoidan in conjunction with chemotherapy for solid tumor patients [145]. In vitro and in-vivo studies imply that *F. vesiculosus* could be a valuable marine drug that warrants additional research and development for clinical use. 

### 3.4. Astragalus membranaceus 

*Astragalus membranaceus*, a popular immunomodulatory herb used in China, has a long history in traditional Chinese medicine. Currently, it is commonly deployed in mixed herbal decoctions as an immunomodulating agent. *Astragalus polysaccharides* have been investigated extensively, especially for their immuno-potentiating features, such as induction of DCs, efficient vaccine adjuvant, and immuno-regulated anticancer activities. *A. membranaceus* polysaccharide raises IL-2, IL-12, and TNF-secretion while lowering IL-10 levels in the blood, conferring anticancer activity in vivo, improving host immune responses, and appears to be safe and effective for antitumor therapy [146]. According to cell viability detection data, *Astragalus polysaccharides* can reduce the proliferation of human lung cancer cell lines A549 and NCI-H358 by suppressing NF-KB transcription activity and down-regulating expression of p65, p50, CyclinD1, and Bcl-xL proteins. Similarly, *Astragalus polysaccharide* has shown up-regulation of p53 and PTEN expression through regulating p53/MDM2 feedback loops, signifying one of the alleged antiproliferative mechanisms [147]. APS activates macrophages and enhances the level of nitric oxide (NO) and tumor necrosis factor-α (TNF-α), both of which serve as pro-apoptotic inducers in MCF- 7 cells [148]. Notably, *Astragalus polysaccharides* have cytotoxic and growth-inhibitory effects against breast cancer by regulating novel molecular therapeutic targets such as EGFR and ANXA1 [149]. In addition, clinical trials have shown that Astragalus polysaccharides can improve the well-being of advanced non-small cell lung cancer (NSCLC) patients by increasing the efficiency of platinum-based chemotherapy [150]. Astragalus polysaccharides, an AKT signaling pathway blocker, can be used in conjunction with Apatinib to treat gastric cancer [151]. Intranasal administration of Astragalus polysaccharide upregulated CCR7 expression, resulting in expansion of CD11C^+^ DCs population and activation NK and T cells in the mesenteric lymph nodes along with growth-inhibitory activity against pulmonary metastatic melanoma in mice. According to in vivo studies, combining *Astragalus polysaccharide* and anti-PD-L1 antibody, immune checkpoint blockade exhibits anti-invasive and antimetastatic effects. These findings suggest the application of *Astragalus polysaccharide* as a topical mucosal adjuvant to boost the anticancer efficacy of immune checkpoint inhibitors [152]. Antioxidant, anti-inflammatory, and proapoptotic *Astragalus polysaccharides* improve the cardiotoxicity of doxorubicin by modulating PI3k/Akt and p38MAPK pathway for high antitumor and antiglomerulonephritis efficacy [153]. Furthermore, *Astragalus polysaccharide* modulates the M1/M2 macrophage pool, enabling DC maturation and synergistically augmenting the anticancer effect of a conventional chemotherapeutic agent, cisplatin. These exploratory investigations set the groundwork for additional research into the curative potential of marine drugs as surrogate immunotherapy and/or the therapeutic viability of using them to treat cancer [154]. *Astragalus polysaccharide* also effectively induced erythroid differentiation in K562 cells by regulating the LMO2, Klf1, Klf3, Runx1, EphB4, and Sp1 genes, promoting γ-globin mRNA expression and fetal hemoglobin synthesis, thereby proving its potential clinical utility for leukemia treatment [155]. Furthermore, in patients with chronic myelogenous leukemia, *Astragalus polysaccharide* improved the immunological activity of plasmacytoid dendritic cells (pDCs) and accelerated their development and maturation [156]. In comparison to 5-fluorouracil (5-FU), Astragalus polysaccharides have high anticancer and immuno-enhancement actions in vivo, involving macrophagic pinocytosis, NK cell killing activity, tumor suppression, and T cell subsets expansion [157]. In line with this, *Astragalusx polysaccharide* alleviates immunosuppression of 5-FU by up-regulating the expression of IL-2, IFN-γ and TNF-α, ensuring adequacy as an immune adjuvant for chemotherapy [158]. Thus, *Astragalus polysaccharides* can be employed for cancer management in combination with conventional chemotherapies to enhance anticancer effects. 

### 3.5. Ascophyllum nodosum

*Ascophyllum nodosum* comprises ascophyllan, fucoidan and alginate. Fucose and xylose are nearly equimolecular in ascophyllan. At the same time, fucoidan had a significantly greater ratio of fucose to xylose, and the concentrations of these monosaccharides in the alginate were extremely low. Regarding in vitro studies, ascophyllan exerts antiproliferative effects by induction of DNA fragmentation, cleavage of PARP, activation of caspases-9 and -3, altered the mitochondrial membrane permeability and typical apoptotic nuclear morphological changes while manifests immune-modulation by stimulating the secretion of TNF-α and granulocyte colony-stimulating factor (G-CSF) [159]. In mice models, intraperitoneal administration of ascophyllan exhibits antimetastatic effects via involving the immune system, specifically enhanced NK cell activity, and inhibiting ERK-mediated MMP-9 activity [160]. Ascophyllan stimulates the ERK, p38, and JNK signaling pathways and acts as an immunostimulant by inducing human monocyte-derived DCs (MDDCs), PBDCs, and blood dendritic cells antigen-BDCA1 and BDCA3 expression [161]. Ascophyllan therapy triggered IFN- γ production and CD69 overexpression and improved NK cell cytotoxicity, but cell proliferation remained unaffected. These findings show that ascophyllan stimulates NK cell activation in vitro and in vivo, and its effect is greater than fucoidan [162]. Nevertheless, several in vitro and in vivo studies imply that ascophyllan exhibit anti-inflammatory effects on stimulated macrophages primarily by reducing NO and ROS generation and activating NK cells in a concentration-dependent manner [163,164]. In general, ascophyllan exerts potential anticancer effects by enhancing immune-enhancing events involving DC activation, Th1 immune response and antibody generation. Hence, ascophyllan can be used as an adjuvant to develop therapeutic and preventive tumor vaccines [165].

### 3.6. Codium fragile

**Figure 2:**
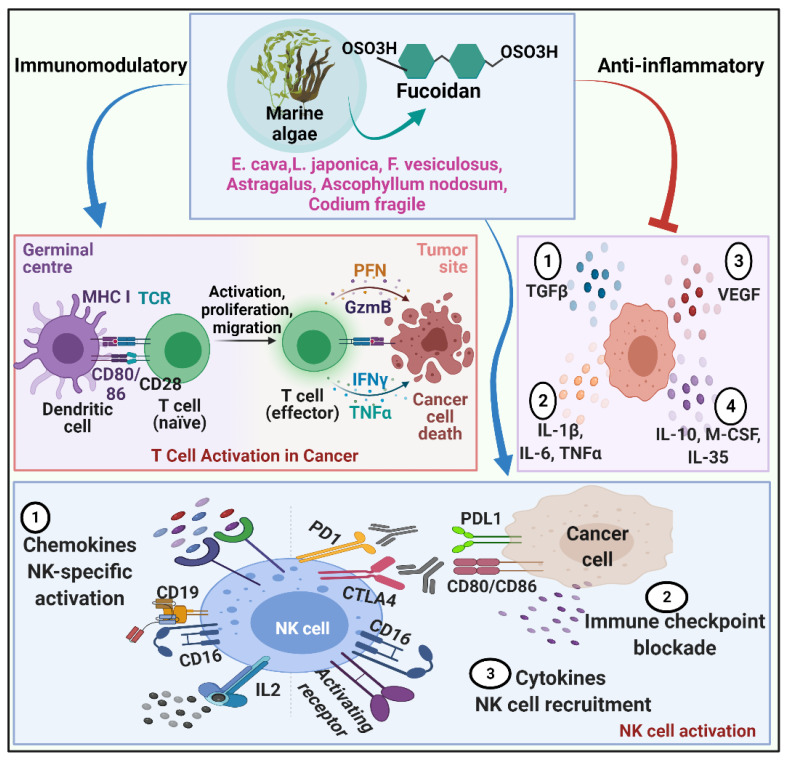
Depicts immunomodulatory features of fucoidan serve as a potential approach to managing cancer metastasis. Fucoidan from marine algae enhances immune cell proliferation, activation and migration. Fucoidan significantly activates NK cells and recruits them into the tumor microenvironment. It also stimulates dendritic cells to activate T cells and modulates immune responses against tumor cells. Fucoidan illustrates anti-inflammatory effects by suppressing the secretion of proinflammatory mediators at the tumor site.

*Codium fragile* (*C. fragile*) polysaccharides are linear homopolymers comprising ß-1.4-linked D-mannose residues. According to research, the anticancer properties of *C. fragile* sulfated polysaccharides are based on the antioxidant and immunomodulatory effects. The antioxidant effects of *C. fragile* sulfated polysaccharides against hydrogen peroxide-mediated oxidative stress in vitro and in vivo models have been extensively reported. In Vero cells, *C. fragile* polysaccharide has shown cytoprotective benefits by reducing intracellular ROS, enhancing cell survival, and regulating apoptosis dose-dependently. In H2O2-stimulated zebrafish, *C. fragile* polysaccharides enhanced survival and restored heartbeat while diminishing ROS, cell mortality, and lipid peroxidation [166]. *C. fragile* polysaccharide improves anticancer immunity by stimulating human monocyte-derived dendritic cells (MDDCs) to secrete more proinflammatory cytokines and overexpress CD80, CD83, and CD86, as well as MHC class I and II. *C. fragile* polysaccharide also induced BDCA1+ and BDCA3+ subsets of PBDCs, promoting syngeneic helper and cytotoxic T cell activation and proliferation [152]. The polysaccharide from C. fragile induces bone marrow-derived dendritic cells (BMDCs) to activate NK cells and infiltrate T lymphocytes that produce IFN and TNF cytokines. *C. fragile* polysaccharide stimulated NK cells, resulting in elevation of cytotoxic mediators such as IFN-γ, perforin, IL-12, granzyme B, and CD69 overexpression, ensuing anticancer immune responses [167]. Furthermore, in the mouse model, combining *C. fragile* polysaccharide and cancer self-antigen therapy effectively reduced B16 tumor growth. C. fragile polysaccharide administration strengthened anti-PD-L1 antibody-mediated anticancer immunity in CT-26 carcinoma-bearing BALB/c mice. These data suggest that *C. fragile* polysaccharide, through increasing immune activation, could be employed as an adjuvant in cancer treatment [167] (Figure 2).

## 4. Challenges and Future Prospects

The battle for life often leads a species to evolve its own distinctive weapons, such as speed, power, or even chemical toxins. These toxins have biologically powerful activity and distinct mechanisms of action, making them potent compounds for further therapeutic study [168,169]. Marine natural products have recently been praised for their exceptional bioactivity in extremely diluted circumstances [170,171]. Consequently, to find and develop new drugs further, it is reasonable to use marine natural products as a hit or lead compound [2,172]. One such strategy was the use of heparanase, a known anticancer drug target for developing anticancer therapy. Heparan sulfate, an essential part of the extracellular matrix, can only be broken down by heparanase, a mammalian enzyme. As a result, the extracellular matrix is remodeled, and growth factors and cytokines bound to heparan sulfate are released. Angiogenesis, immune cell migration, inflammation, wound healing, and metastasis is a few examples of physiological and pathological processes that are subsequently promoted [173]. Numerous studies have shown conclusively that heparanase plays a critical role in the advancement of cancer by degrading HSPGs, which in turn promotes neovascularization processes that support the invasion of metastatic cells [174,175]. Substances with a high level of heparanase inhibition and a low level of extracellular matrix-bound growth factor release or potentiation may hold hope for their potential as antiangiogenic and antimetastatic therapies [176]. Heparin and its derivatives, as well as other sulfated polysaccharides like fucoidan, have heparanase inhibitory activity, which enables them to block cancer cell invasion or prevent metastasis in in vitro experiments [177,178]. Although marine natural products offer a high potential for drug discovery, there are a few challenges associated with them. Despite extensive studies on fucoidan’s anticancer potential and targeting capabilities, the use of fucoidan as a cancer treatment molecule or a drug delivery system has hit certain roadblocks. Firstly, pure fucoidan is indeed a costly and limited biomolecule as it is a natural polymer obtained from brown seaweed. It is difficult to obtain a sufficient quantity of these chemicals for future research [179]. Also, extraction and purification operations are sophisticated and involve several painstaking steps. *Fucus vesiculosus*, *Macrocystis pyrifera*, *Laminaria japonica* and *Undaria pinnatifida* have all been commercially marketed fucoidans. Due to the apparent expensive cost, fucoidan applications for research may have been limited, and its industrial application may have been held back. Secondly, another challenge is structural complexity. The monosaccharide content, molecular weight, and sulfation degree of fucoidan from various sources differ [180]. Due to its intricate structure, the manipulation or synthesis of marine natural products and the development of a less expensive synthetic mimic of fucoidan have been challenged [181,182]. Biological activity and its selectivity is the third obstacle. Since marine natural products are originated from marine species rather than mammals, their administration may cause undesirable biological processes in humans [183]. Fourthly, safety analysis and biodegradability testing are underway. Fucoidan has been eventually a food ingredient consumed for a long period by Asians. Even though fucoidan has been used as a food and dietary supplement for decades, studies have been undertaken to determine the safety and estimate hepatotoxicity and nephrotoxicity of pure extract and LMW fucoidan. The safety profile of fucoidan has been evaluated in vivo acute toxicity trials (4 weeks), and no significant adverse effects have been observed. Furthermore, in murine models, fucoidan from *F. vesiculosus*, *Laminaria japonica*, and *Undaria pinnatifida* exhibited no toxicological manifestations [184,185,186]. However, fucoidan is considered to be eliminated intact in urine since it is not a biodegradable polymer. Researchers must develop a consensus on the various families of medications that can be derived from marine algae, their mechanisms of action, and the possibilities of offering these phytochemical substances as dietary supplements to cancer patients [187]. Fifthly, the safety of fucoidan, whether utilized as nanoparticles or as a coating material for various nanosystems, has yet to be determined. In order to confirm its use as a safe and effective anticancer agent, more studies should be conducted to test the long-term safety of fucoidan nanoparticles.

Nowadays, studies are urged to support the potential evaluation of fucoidan in prospective clinical trials in combination with chemotherapy to improve cancer patient outcomes. Several preclinical investigations have already been conducted to evaluate the potential hepatic metabolism-mediated chemo-drug interactions with fucoidan. Fucoidan from *F. vesiculosus* demonstrated considerable synergistic influence when combined with paclitaxel and tamoxifen [145], whereas it had additive efficacy with topotecan and no antagonistic activity with letrozole [188]. Hence, *F. vesiculosus* fucoidan may have minimal drug-supplement interactions with either the CYP450 or the COMT hepatic metabolic pathways. Notably, fucoidan does not support tumor growth, and its immunomodulatory effect may inhibit tumor growth; however, this evidence needs to be validated in future clinical trials. Future research is needed to corroborate the findings of fucoidans in combination with chemotherapy [188,189].

Fucoidan is a potential biopolymer with vivid applications in cancer treatment. The polymer contains inherent anticancer qualities, as well as excellent drug delivery characteristics, and active targeting capabilities. An excellent application for fucoidan is cancer-targeted therapy. Fucoidan can be conjugated to cancer-specific target molecules or chemo drugs or encased in liposomes that release their payload exclusively in neoplastic cells. However, before clinical trials, the inclusive structure and other chemical features of fucoidan must be determined. Most studies on polysaccharide nanoparticles in the literature employ quite a common carrier material such as Chitosan (CS), polyethyleneimine (PEI), polyamidoamine (PAMAM), and poly-l-lysine (PLL), epirubicin (EPI)-carrageenan oligosaccharide (CAO)-gold (Au) nanoparticles for nano delivery. Furthermore, well-designed clinical trials are required to examine the effectiveness and safety of marine drugs in cancer patients.

## 5. Conclusions

Marine drugs are key in cancer care and can be regarded as future frontiers in pharmaceutical research. The anticancer mechanism shown by marine drugs commonly involves regulation of signal transduction, cell cycle arrest, cell apoptosis, and inhibition of migration and neo-angiogenesis, and also stimulates the immune responses and antioxidant system to prevent cancer. Maintenance of immune homeostasis and regulation are significant in cancer treatment, and according to recent research, immunomodulatory marine drugs have a high potential to reduce the negative effects of immunosuppressive chemotherapies. The pathophysiological pathways which play an imperative role in the therapeutic and preventative activities of marine medications against oncogenesis are VEGF/VEGFR2, TLR4/ROS/ER, TGFR/Smad7/Smurf2, PI3K/AKT/mTOR, p38/MAPK/ERK/JNK, TGFR/Smad/Snail, β-catenin/wnt, CXCL12/ CXCR4, and PBK/topk. Marine polysaccharides primarily produce antitumor effects by direct cytotoxicity, immune stimulation, and synergistic effects with conventional anticancer medicines. Since fucoidan is a natural polymer, it requires extensive processing before medicinal use, and the structure of the extracted polymer is highly reliant on the source. The considerations above may limit its use on a large basis. As a result, more research is needed to establish strong structure-activity connections. Further exploration is therefore recommended to develop a solid structural activity correlation that paves the way for a polymer synthesis with expected activity and possible safety. Conclusively, fucoidan is an invaluable multifunctional therapeutic drug against cancer.

## Figures and Tables

**Table 1:** Case studies of fucoidan in alternative medicine and complementary therapy: human clinical trials.

Fucoidan Source	Cancer Type	Results	Risk of Clinically Significant Interactions	Reference
*U. pinnatifida*-	Breast cancer	No significant changes	Absent	[95]
Low-molecular-weight fucoidan (LMF)	Metastatic colorectal cancer (mcrc)	Improved disease control rate	Absent	[96]
HiQ-fucoidan from *Laminaria japonica*	Lung cancer	Survival rates increased by approx. 50%	Reduced the occurrence of general fatigue	[59]
*Cladosiphon okamuranus*	Colorectal cancer	Endure prolonged chemotherapy without fatigue	-Suppressed general fatigue -No suppression of diarrhea and neurotoxicity	[97]
Mozuku, *Cladosiphon novae-caledoniae* Kylin	Advanced cancer	Decreased level of proinflammatory cytokines	Insignificant quality of life score	[98]

**Table 2:** In-vivo effect of fucoidan on tumor growth in tumor-bearing mice.

Fucoidan Source	Cancer Cell Type	Action Mechanism	Reference
*Cladosiphon okamuranus*	Colon 26	tumor growth↓	[99]
(LMWF)			
IMWF and HMWF		↑survival time	
Oral administration		↑NK cells in the spleen	
*Fucus vesiculosus*	4T1 Lewis lung cancer cells B16	-Inhibition of angiogenesis -Induction of apoptosis -Prevention of metastasis	[59,78,79]
*Fucus evanescence*	Lewis lung cancer cells	Antitumor and antimetastatic activities	[63]
*Sargassum plagiophyllum*	Diethylnitrosamine-induced hepatocellular	Inhibition of carcinogen metabolism	[100]
*Cladosiphon okamuranus Tokida*	Sarcoma 180 (S-180)-xenograft	↑cytotoxicity via NO production by fucoidan-stimulated macrophages	[101]
*Undaria pinnatifida*	A20	Cytolytic activity by Th1 and NK cell activation	[102]
*Ascophyllum nodosum*	MOPC-315 plasma cell tumor	Anti-angiogenesis	[103]
*Sargassum mcclurei*	colon cancer DLD-1 cells	Anti-tumorigenesis	[104]
*Ecklonia cava*	SKOV3 tumor xenograft	-↑ROS-mediated apoptosis -antitumoral	[105]
	CT-26 carcinoma xenograft	↑NK cell-mediated anticancer immunity	[106]
*Hydroclathrus clathratus*	Sarcoma 180 xenograft	Suppressed tumor growth	[107]
*Sargassum stenophyllum*	B16F10 cells	Antiangiogenic and antitumoral	[108]
*Stoechospermum marginatum*	Ehrlich ascites tumor (EAT) cells	Angio-suppressive and antiproliferative activities	[109]
*Sargassum fusiforme*	A549	Immunomodulatory activity	[110]
	SPC-A-1	Anti-angiogenesis	[111]

**Table 3:** In-vitro effect of fucoidan on different cancer cell types.

Fucoidan Source	Cell Type	Action Mechanism	Action Characteristic	Reference
*Sargassum pallidum*	HepG2, A549, and MGC-803	Antitumor activity	-Antioxidant	[112]
*Sargassum tortile*	P-388	Increases cytotoxicity	-	[113]
*Sargassum micracanthum*	Human head and neck squamous cell carcinoma (HNSCC)	Anticancer efficacy	-	[114]
*Undaria pinnatifida*	A549 SMMC-7721 NB4, KG1a, HL60, and K562	-Induces apoptosis -Inhibit cell proliferation	Down-regulation of p38, PI3K/Akt, and the activation of the ERK1/2 MAPK pathway -NK-cell ↑ -Livin, XIAP mRNA ↓ Caspase-3,8,9 ↑ Bax-to-Bcl-2 ratio↑ Cytochrome c ↑ -ROS↑	[40,115]
*Fucus vesiculosus*	HT-29 MCF-7 MDA-MB-231 Lewis lung A549 H1975 Huh-7 SNU-761 SNU-3085 HL-60 NB4 THP-1 SUDHL-4 OCI-LY8 NU-DUL-1 TMD8 U293 DB	-Inhibit cell proliferation -Induce cell apoptosis -Inhibit metastasis	-IRS-1/PI3K/AKT↓ -Ras/Raf/ERK↓ -Caspase-7,8,9 activation, cytochrome c, Bax ↑ Bcl-2↓ -Smad2/3,Smad4↓ -NF-κB↓ -Inhibit VEGF,MMPs -Caspase-3↑ PARP cleavage -ERK1/2, MEK1/2, JNK ↑	[59,70,116,117]
*Sargassum macrocarpum*	MDA-MB-231, A549, and HCT116	Induces ROS-mediated apoptosis	Inhibits STAT3 Signaling	[118]
*Sargassum muticum*	MCF-7 and MDA-MB-231	Induce apoptosis, antioxidant, and antiangiogenesis effects	-	[119]
*Sargassum angustifolium*	HeLa and MCF-7	Cytotoxic activity	-	[120]
*Sargassum cinereum*	Caco-2 and HCT-15	Anticancer and apoptotic effect	Enhances ROS production	[121,122]
*Sargassum filipendula*	HeL	Induces apoptosis	Down-regulates Bcl-2	[123]
*Ecklonia cava*	CT-26	Induces apoptosis	Bcl-2/Bax signal pathway	[68]
*Eisenia bicyclis*	SK-MEL-28, DLD-1	Inhibited the colony formation	-	[124,125]
*Hizikia fusiformis*	PC3	Induces ROS-dependent apoptosis	Elevated expression of Fas, FasL, Bax and tBid, and decreased expression of Bcl-2 -reduced c-Flip expression and activated caspase-8, -9 and -3, leading to an increment of poly (ADP-ribose) polymerase (PARP) cleavage	[126]
*Hydroclathrus clathratus*	HL-60, MCF-7	Antiproliferative activity	Induced sub-G1 arrest	[127]
*Saccharina*	DLD-1	Inhibit cell proliferation	Inhibit the binding of EGF receptor with EGF	[38]
	T-47D			
*Sargassum*	DLD-1 Huh6 Huh7 SK-Hep1 HepG2	Inhibit cell proliferation	-Colony formation inhibition -TGF-β R1, 2↓ Phospho-Smad2/3↓ Smad 4 protein↓	[38]
*Cladosiphon*	MCF-7	Induce cell apoptosis	PARP cleavage Caspase-7,8,9 ↑ Cytochrome C, Bax, Bid↑	[38]
*Bifurcaria bifurcata*	NSCLC-N6	Inhibit cell proliferation	The growth arrest is irreversible	[38]
*Turbinaria conoides*	A549	-Inhibit cell proliferation -Induce cell apoptosis	G0/G1 phase arrest	[38]
*Sargassum latifolium*	leukemia (1301 cells)	Chemopreventive activity	Antioxidant capacity	[128]
*Sargassum glaucescens*	MCF-7 HT-29	Induce apoptosis	Fragmented the DNA of cancer cells	[129]

Upwards arrow shows an increase (↑), a downwards arrow a decrease (↓).
